# Supplementary material for: Association of complement C3 inhibitor pegcetacoplan with reduced photoreceptor degeneration beyond areas of geographic atrophy
Source: Sci Rep. 2022 Oct 25;12:17870. doi: 10.1038/s41598-022-22404-9 (PMC9596427; doi:10.1038/s41598-022-22404-9)
Supplement: Supplementary file 1 — Supplementary Information. [file 41598_2022_22404_MOESM1_ESM.docx]

**Supplementary Information**

Pfau M, Schmitz-Valckenberg S, Ribeiro R, et al. Association of Complement C3 Inhibitor Pegcetacoplan with Reduced Photoreceptor Degeneration Beyond Areas of Geographic Atrophy

|  | Page |
| --- | --- |
| **Supplementary Methods.** Image segmentation | 2 |
| **Supplementary Figure S1.** Clinical trial flowchart | 3 |
| **Supplementary Figure S2.** Accuracy of the retinal pigment epithelium (RPE) atrophy segmentation in study eyes | 4 |
| **Supplementary Figure S3.** Study eye thickness change at the level of photoreceptor inner segments (IS) and outer segments (OS) over time | 5 |
| **Supplementary Figure S4.** Study eye thickness changes at the level of the photoreceptor layers over time along three contour-lines (modified intention-to-treat [mITT] analysis) | 6 |
| **Supplementary Figure S5.** Study eye thickness changes at the level of the photoreceptor layers over time along three contour-lines (per-protocol [PP] analysis) | 7 |
| **Supplementary Figure S6.** Study eye thickness changes at the level of the photoreceptor layers over time along three contour-lines (per-protocol [PP] analysis, excluding eyes with exudative macular neovascularization [MNV]) | 8 |
| **Supplementary Figure S7.** Fellow eye thickness changes at the level of the photoreceptor layers over time along three contour-lines (modified intention-to-treat [mITT] analysis) | 9 |
| **Supplementary Table S1.** Comparison of the baseline cohort characteristics of the included (Spectralis imaging) and excluded (Cirrus imaging) patients | 10 |
| **Supplementary Table S2.** Study eye differences in thickness at the level of photoreceptor layers (in z-score units) along three contour-lines at month 12 (per-protocol [PP] analysis) | 11 |
| **Supplementary Table S3.** Study eye differences in thickness at the level of photoreceptor layers (in z-score units) along three contour-lines at month 12 (per-protocol [PP] analysis, excluding eyes with exudative macular neovascularization [MNV] at any visit) | 12 |
| **Supplementary Table S4.** Fellow eye differences in thickness at the level of photoreceptor layers (in z-score units) along three contour-lines at month 12 (modified intention-to-treat [mITT] analysis) | 13 |
| **Supplementary Table S5.** Availability of SD-OCT data | 14 |
| **Online-Only References** | 15 |

**Supplementary Methods. Image segmentation**

Using a first (‘layer segmentation’) CNN,^1^ we segmented the (1.) inner retina (internal limiting membrane [ILM] to the outer plexiform layer/outer nuclear layer boundary [OPL/ONL]), (2.) ONL including Henle’s fiber layer (HFL+ONL, OPL/ONL to the external limiting membrane [ELM]), (3.) the photoreceptor inner segments (IS, ELM to the ellipsoid zone [EZ]), (4.) the photoreceptor outer segments (OS, EZ to the upper boundary of the RPE-drusen complex [RPEDC]), (5.) the RPEDC, and (6.) the choroid. The RPEDC included reticular pseudodrusen (a.k.a. SDD), and the RPE, drusen. The outer boundary for the RPEDC was Bruch’s membrane (BrM). Henle’s fiber layer was counted toward the ONL in consideration of reproducibility.^2^ For the en-face segmentation of the area of GA, peri-papillary atrophy (PPA), and the optic nerve head (ONH), we applied a second – also previously validated - ‘en-face’ segmentation CNN.^1^

**Supplementary Figure S1. Clinical trial flowchart**

The modified intention-to-treat (mITT) population included patients with at least one injection and at least one follow-up examination at month 2 (or later) at which efficacy data were collected.

Supplementary Table S5 provides a detailed overview for each arm and visit of the image data that could be included in the analyses.

***Abbreviations:*** *every other month (EOM), intention-to-treat (ITT), modified ITT (mITT), spectral-domain optical coherence tomography (SD-OCT)*

**
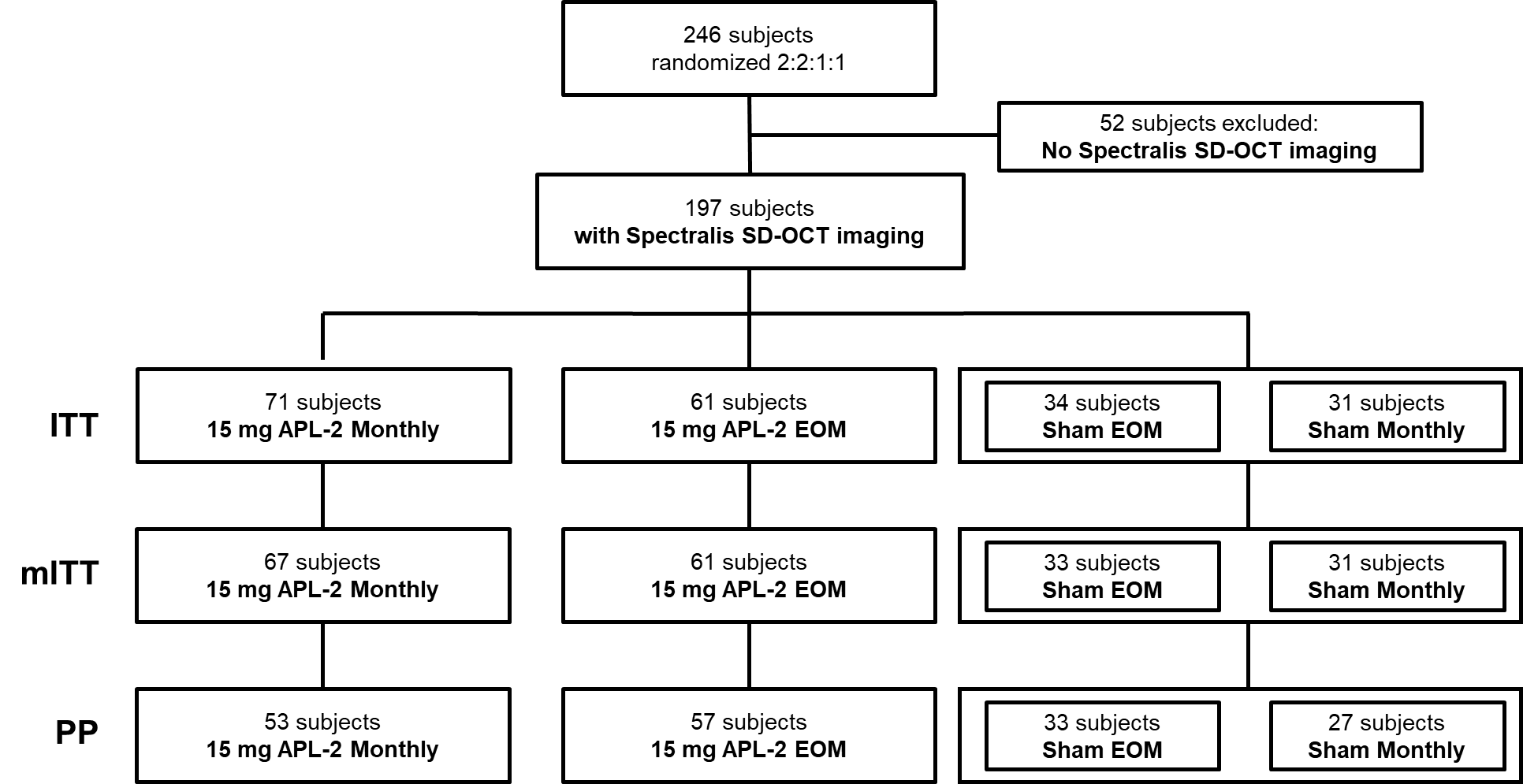
**

**Supplementary Figure S2. Accuracy of the retinal pigment epithelium (RPE) atrophy segmentation in study eyes**

This analysis included only visits without RPE-atrophy exceeding the optical coherence tomography (OCT) image frame of 20°x20°. Panel A shows the correlation of the implemented OCT-based analysis using deep-learning (DL) with the original, manually-annotated fundus autofluorescence (FAF)-based square-root transformed area of RPE-atrophy. Panel B shows the corresponding Bland–Altman plot with the mean difference (bias, solid red line) and 95% limits of agreement (red, dashed lines). Both of these lines were estimated using mixed model analysis. There was no statistically significant bias between these methods (bias estimate [95% CI] of 0.02 mm [0.00; 0.04]). Patients were treated between baseline and month 12. Panel C shows the least-squared (adjusted) means and 95% confidence intervals for the progression of RPE-atrophy over time.

***Abbreviations:*** *retinal pigment epithelium (RPE), fundus autofluorescence (FAF), deep learning (DL)*


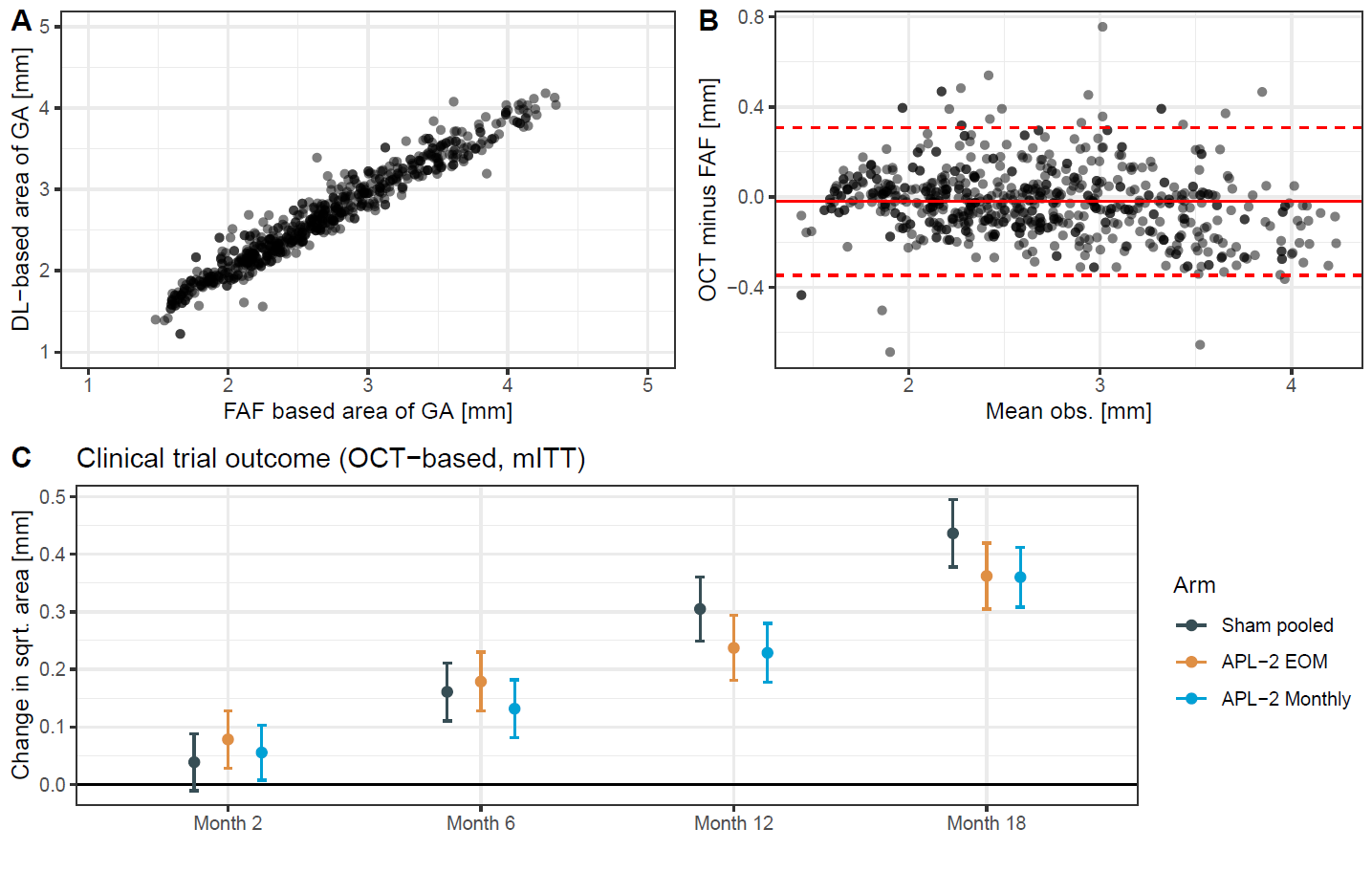


**Supplementary Figure S3. Study eye thickness change at the level of photoreceptor inner segments (IS) and outer segments (OS) over time**

The plots show the average photoreceptor inner segments (IS) and outer segments (OS) thickness and standard error of the mean (SEM, ribbons) with the distance to the boundary of retinal pigment epithelium (RPE) atrophy (x-axis). The vertical dashed lines denote the 0.43°, 2.58°, and 5.16° contour-lines that were considered for the linear mixed model analyses. Patients were treated between baseline and month 12.

The panels indicate the visit, and the color indicates the treatment arm. The data were derived from the modified intention-to-treat (mITT) analysis (N_patients_= 192).

***Abbreviations:*** *outer nuclear layer (ONL), photoreceptor inner segments (IS), photoreceptor outer segments (OS), modified intention-to-treat (mITT)*


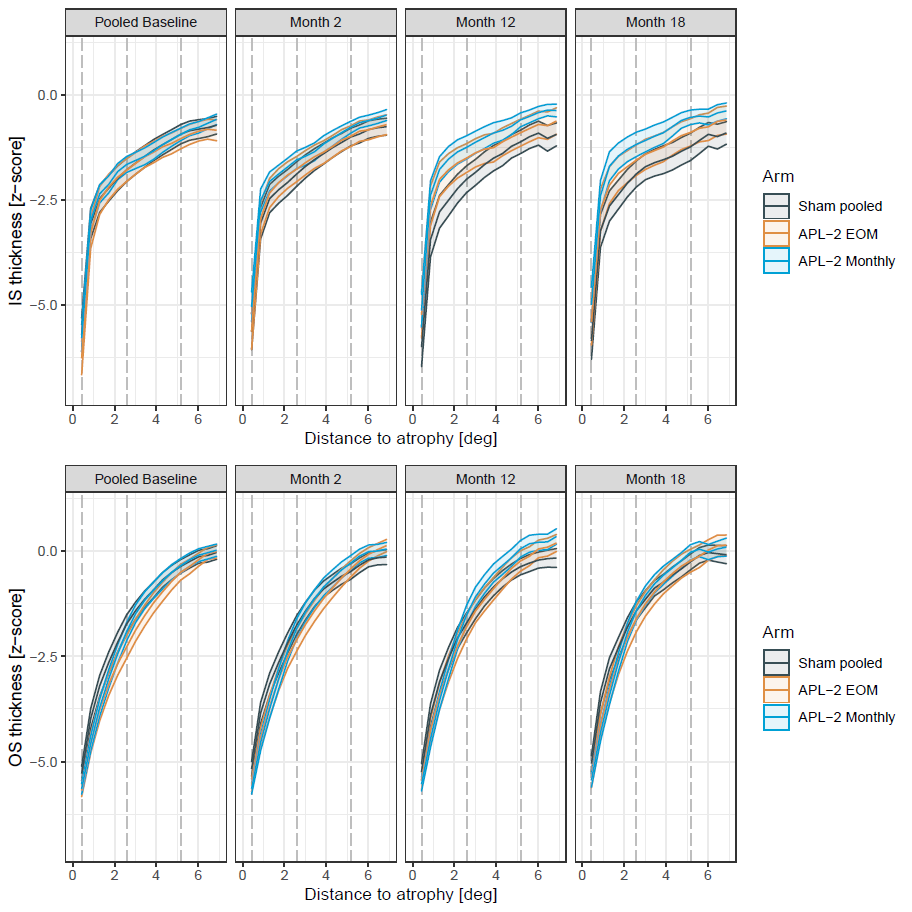


**Supplementary Figure S4. Study eye thickness changes at the level of the photoreceptor layers over time along three contour-lines (modified intention-to-treat [mITT] analysis)**

The plots show the least-squares (adjusted) means from the linear mixed model analysis for the outer nuclear layer (ONL), photoreceptor inner segments (IS), and photoreceptor outer segments (OS) thickness (rows) along all three contour-line (columns) in dependence of the visit (x-axis) and treatment arm (colors). The lines denote the 95% confidence intervals. Eyes treated with pegcetacoplan (APL-2) monthly tended to show over time a lesser degree of photoreceptor laminae thinning in the junctional zone. Patients were treated between baseline and month 12. Please note that the last column of this figure (i.e., 5.16° mITT contour analysis) shows the same data as Figure 3 in the main text.

***Abbreviations:*** *outer nuclear layer (ONL), photoreceptor inner segments (IS), photoreceptor outer segments (OS), modified intention-to-treat (mITT)*


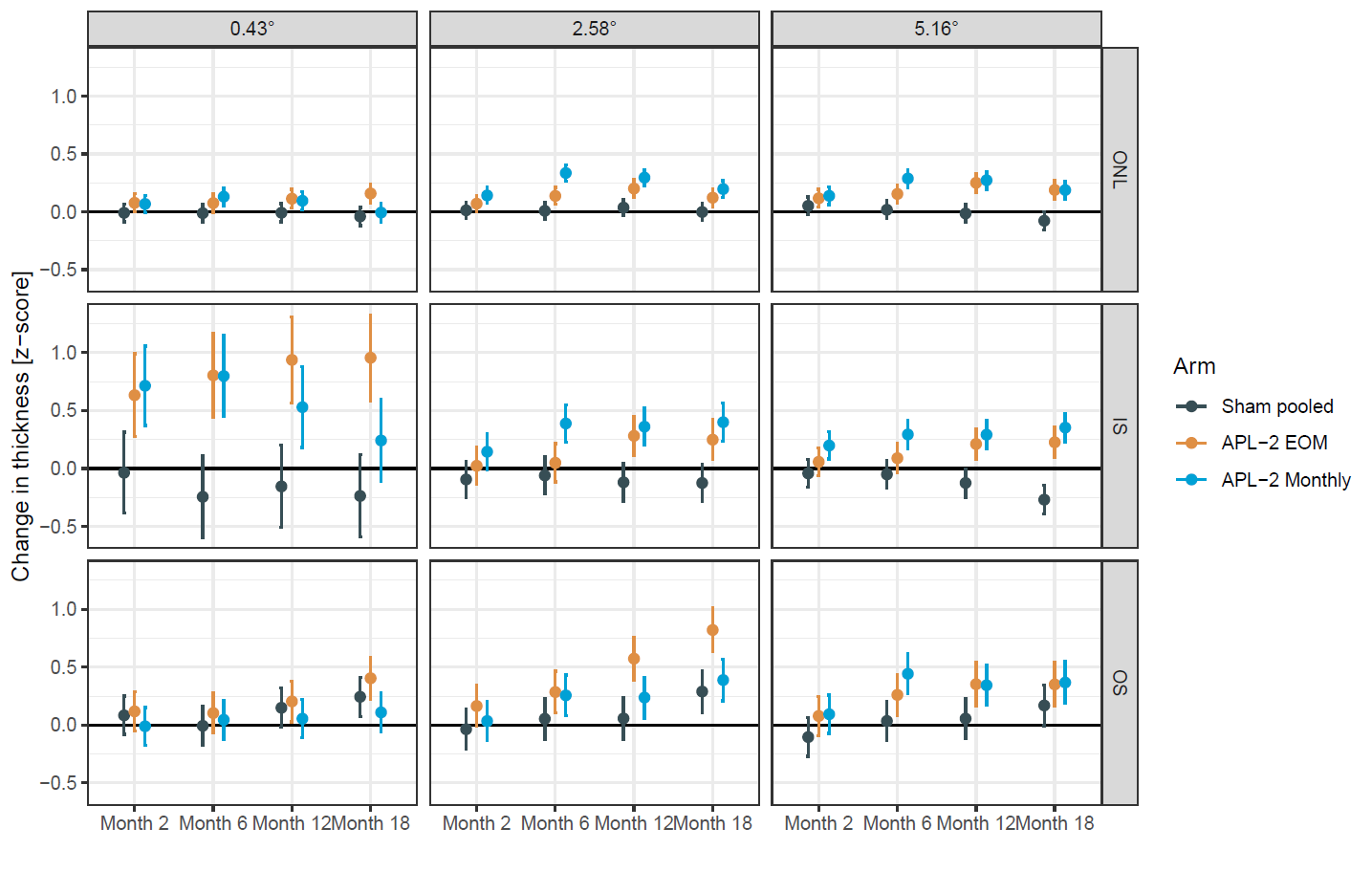


**Supplementary Figure S5. Study eye thickness changes at the level of the photoreceptor layers over time along three contour-lines (per-protocol [PP] analysis)**

The plots show the least-squares (adjusted) means from the linear mixed model analysis for the outer nuclear layer (ONL), photoreceptor inner segments (IS), and photoreceptor outer segments (OS) thickness (rows) along all three contour-line (columns) in dependence of the visit (x-axis) and treatment arm (colors). The lines denote the 95% confidence intervals. Eyes treated with pegcetacoplan (APL-2) monthly tended to show over time a lesser degree of photoreceptor laminae thinning in the junctional zone. Patients were treated between baseline and month 12.

***Abbreviations:*** *outer nuclear layer (ONL), photoreceptor inner segments (IS), photoreceptor outer segments (OS), per-protocol (PP)*


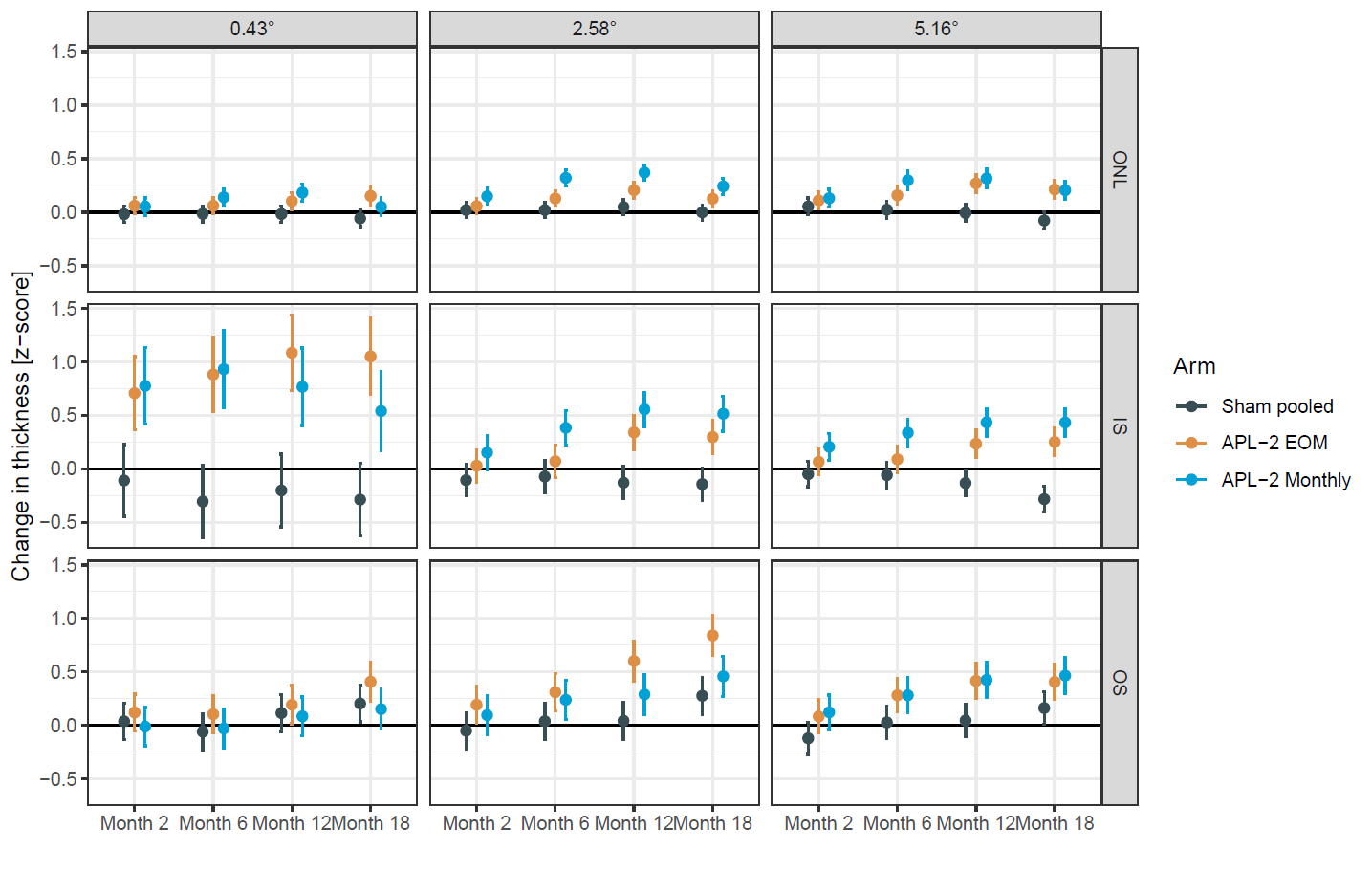


**Supplementary Figure S6. Study eye thickness changes at the level of the photoreceptor layers over time along three contour-lines (per-protocol [PP] analysis, excluding eyes with exudative macular neovascularization [MNV])**

The plots show the least-squares (adjusted) means from the linear mixed model analysis for the outer nuclear layer (ONL), photoreceptor inner segments (IS), and photoreceptor outer segments (OS) thickness (rows) along all three contour-line (columns) in dependence of the visit (x-axis) and treatment arm (colors). The lines denote the 95% confidence intervals. Eyes treated with pegcetacoplan (APL-2) monthly tended to show over time a lesser degree of photoreceptor laminae thinning in the junctional zone. Patients were treated between baseline and month 12.

For this sensitivity analysis, all visits were excluded from eyes that exhibited exudative MNV at any point in time.

***Abbreviations:*** *outer nuclear layer (ONL), photoreceptor inner segments (IS), photoreceptor outer segments (OS), per-protocol (PP), macular neovascularization (MNV)*


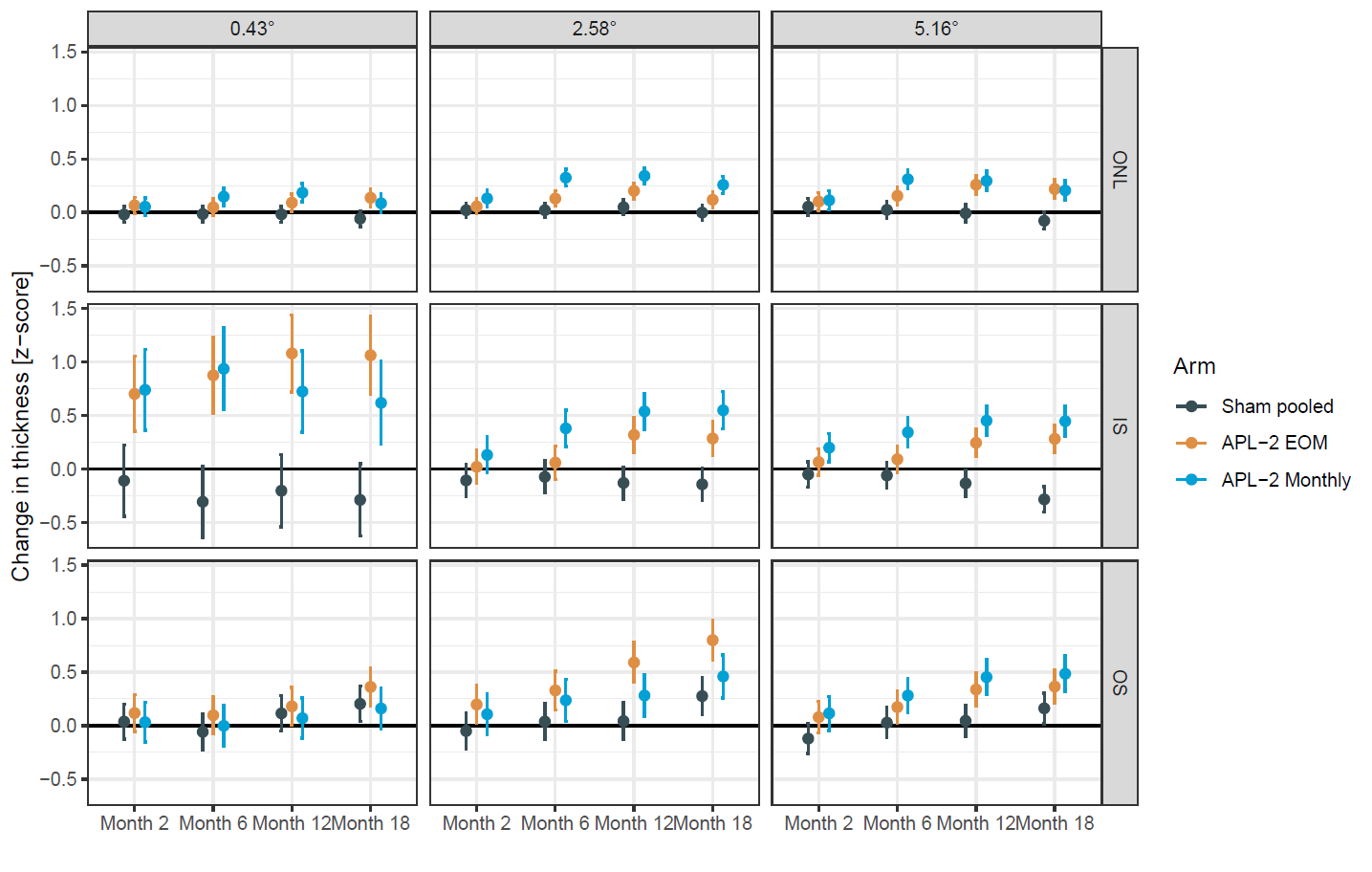


**Supplementary Figure S7. Fellow eye thickness changes at the level of the photoreceptor layers over time along three contour-lines (modified intention-to-treat [mITT] analysis)**

The plots show the least-squares (adjusted) means from the linear mixed model analysis for the outer nuclear layer (ONL), photoreceptor inner segments (IS), and photoreceptor outer segments (OS) thickness (rows) along all three contour-line (columns) in dependence of the visit (x-axis) and treatment arm (colors). The lines denote the 95% confidence intervals. Eyes treated with pegcetacoplan (APL-2) monthly tended to show over time a lesser degree of photoreceptor laminae thinning in the junctional zone. Patients were treated between baseline and month 12.

***Abbreviations:*** *outer nuclear layer (ONL), photoreceptor inner segments (IS), photoreceptor outer segments (OS), modified intention-to-treat (mITT)*


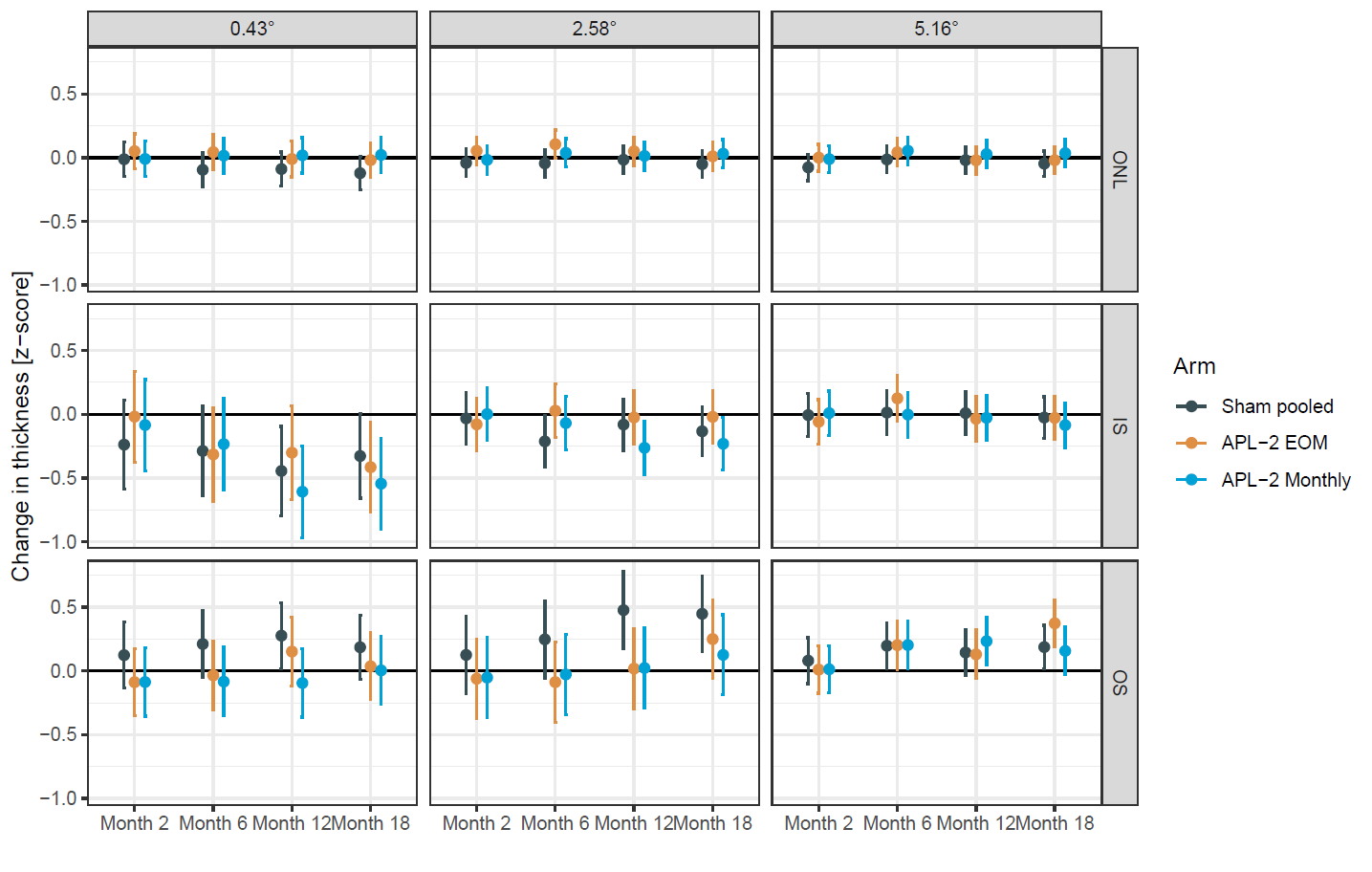


**Supplementary Table S1. Comparison of the baseline cohort characteristics of the included (Spectralis imaging) and excluded (Cirrus imaging) patients**

***Abbreviations:*** *retinal pigment epithelium (RPE), square-root transformed (SQRT)*

|  | **Cirrus** | | | **Spectralis** | | |
| --- | --- | --- | --- | --- | --- | --- |
|  | **Sham pooled**  **(N=16)** | **APL-2 Monthly**  **(N=15)** | **APL-2 EOM**  **(N=17)** | **Sham pooled**  **(N=64)** | **APL-2 Monthly**  **(N=67)** | **APL-2 EOM**  **(N=61)** |
| **Age in years** |  |  |  |  |  |  |
| Mean (SD) | 80.6 (6.61) | 76.1 (6.99) | 83.3 (7.71) | 78.0 (7.62) | 80.1 (7.44) | 80.3 (7.43) |
| Median [Min, Max] | 79.0 [72.0, 94.0] | 77.0 [64.0, 88.0] | 83.0 [68.0, 94.0] | 78.0 [60.0, 96.0] | 81.0 [63.0, 95.0] | 80.0 [60.0, 97.0] |
| **Sex** |  |  |  |  |  |  |
| F | 10 (62.5%) | 10 (66.7%) | 9 (52.9%) | 38 (59.4%) | 44 (65.7%) | 40 (65.6%) |
| M | 6 (37.5%) | 5 (33.3%) | 8 (47.1%) | 26 (40.6%) | 23 (34.3%) | 21 (34.4%) |
| **SQRT-transformed area of RPE-atrophy in mm** |  |  |  |  |  |  |
| Mean (SD) | 2.73 (0.743) | 2.77 (0.716) | 2.95 (0.855) | 2.78 (0.725) | 2.75 (0.663) | 2.88 (0.772) |
| Median [Min, Max] | 2.75 [1.65, 3.89] | 2.65 [1.73, 4.12] | 3.31 [1.61, 4.03] | 2.65 [1.60, 4.07] | 2.74 [1.59, 4.16] | 3.00 [1.59, 4.12] |

**Supplementary Table S2. Study eye differences in thickness at the level of photoreceptor layers (in z-score units) along three contour-lines at month 12 (per-protocol [PP] analysis)** ***

|  |  | **0.43° contour-line** | | | **2.58° contour-line** | | | **5.16° contour-line** | | |
| --- | --- | --- | --- | --- | --- | --- | --- | --- | --- | --- |
| **Layer** | **Contrast** | **Estimate** | **95% CI** | **P-value** | **Estimate** | **95% CI** | **P-value** | **Estimate** | **95% CI** | **P-value** |
| ONL | (APL-2 Monthly) - Sham pooled | 0.2 | [0.07, 0.33] | .001 | 0.32 | [0.2, 0.44] | <.001 | 0.32 | [0.18, 0.46] | <.001 |
| ONL | (APL-2 EOM) - Sham pooled | 0.12 | [-0.01, 0.25] | .08 | 0.16 | [0.04, 0.28] | .007 | 0.28 | [0.14, 0.42] | <.001 |
| ONL | (APL-2 Monthly) - (APL-2 EOM) | 0.08 | [-0.06, 0.21] | .35 | 0.17 | [0.04, 0.29] | .005 | 0.05 | [-0.1, 0.19] | .73 |
| IS | (APL-2 Monthly) - Sham pooled | 0.97 | [0.37, 1.56] | <.001 | 0.69 | [0.42, 0.95] | <.001 | 0.57 | [0.36, 0.78] | <.001 |
| IS | (APL-2 EOM) - Sham pooled | 1.29 | [0.7, 1.87] | <.001 | 0.47 | [0.21, 0.73] | <.001 | 0.37 | [0.16, 0.58] | <.001 |
| IS | (APL-2 Monthly) - (APL-2 EOM) | -0.32 | [-0.92, 0.29] | .43 | 0.22 | [-0.05, 0.48] | .14 | 0.2 | [-0.02, 0.41] | .07 |
| OS | (APL-2 Monthly) - Sham pooled | -0.03 | [-0.33, 0.27] | .97 | 0.25 | [-0.06, 0.55] | .13 | 0.38 | [0.11, 0.65] | .003 |
| OS | (APL-2 EOM) - Sham pooled | 0.08 | [-0.22, 0.37] | .81 | 0.56 | [0.26, 0.86] | <.001 | 0.37 | [0.1, 0.64] | .003 |
| OS | (APL-2 Monthly) - (APL-2 EOM) | -0.11 | [-0.41, 0.2] | .68 | -0.31 | [-0.62, 0] | .05 | 0.01 | [-0.27, 0.28] | 1 |

** P-values were obtained using Kenward-Roger approximation to estimate the denominator degrees of freedom. P-values were adjusted within each model (i.e., the combination of layer and contour-line) using the Tukey method for comparing a family of 3 estimates.*

**Supplementary Table S3. Study eye differences in thickness at the level of photoreceptor layers (in z-score units) along three contour-lines at month 12 (per-protocol [PP] analysis, excluding eyes with exudative macular neovascularization [MNV] at any visit) ***

|  |  | **0.43° contour-line** | | | **2.58° contour-line** | | | **5.16° contour-line** | | |
| --- | --- | --- | --- | --- | --- | --- | --- | --- | --- | --- |
| **Layer** | **Contrast** | **Estimate** | **95% CI** | **P-value** | **Estimate** | **95% CI** | **P-value** | **Estimate** | **95% CI** | **P-value** |
| ONL | (APL-2 Monthly) - Sham pooled | 0.2 | [0.34, 0.07] | .001 | 0.29 | [0.42, 0.17] | <.001 | 0.3 | [0.45, 0.16] | <.001 |
| ONL | (APL-2 EOM) - Sham pooled | 0.11 | [0.24, -0.02] | .12 | 0.15 | [0.27, 0.03] | .009 | 0.27 | [0.41, 0.13] | <.001 |
| ONL | (APL-2 Monthly) - (APL-2 EOM) | 0.09 | [-0.04, 0.23] | .25 | 0.14 | [0.01, 0.27] | .03 | 0.04 | [-0.11, 0.18] | .84 |
| IS | (APL-2 Monthly) - Sham pooled | 0.93 | [1.54, 0.32] | .001 | 0.67 | [0.94, 0.4] | <.001 | 0.59 | [0.8, 0.37] | <.001 |
| IS | (APL-2 EOM) - Sham pooled | 1.28 | [1.88, 0.69] | <.001 | 0.45 | [0.71, 0.19] | <.001 | 0.38 | [0.59, 0.17] | <.001 |
| IS | (APL-2 Monthly) - (APL-2 EOM) | -0.36 | [-0.98, 0.27] | .38 | 0.22 | [-0.06, 0.5] | .16 | 0.21 | [-0.02, 0.43] | .08 |
| OS | (APL-2 Monthly) - Sham pooled | -0.04 | [0.25, -0.34] | .93 | 0.24 | [0.55, -0.07] | .17 | 0.41 | [0.67, 0.15] | <.001 |
| OS | (APL-2 EOM) - Sham pooled | 0.07 | [0.36, -0.23] | .86 | 0.55 | [0.85, 0.24] | <.001 | 0.29 | [0.55, 0.04] | .02 |
| OS | (APL-2 Monthly) - (APL-2 EOM) | -0.11 | [-0.42, 0.2] | .68 | -0.31 | [-0.63, 0.01] | .06 | 0.12 | [-0.15, 0.38] | .57 |

** P-values were obtained using Kenward-Roger approximation to estimate the denominator degrees of freedom. P-values were adjusted within each model (i.e., the combination of layer and contour-line) using the Tukey method for comparing a family of 3 estimates.*

*For this sensitivity analysis, all visits were excluded from eyes that exhibited exudative MNV at any point in time.*

**Supplementary Table S4. Fellow eye** **differences in thickness at the level of photoreceptor layers (in z-score units) along three contour-lines at month 12 (modified intention-to-treat [mITT] analysis) ***

|  |  | **0.43° contour-line** | | | **2.58° contour-line** | | | **5.16° contour-line** | | |
| --- | --- | --- | --- | --- | --- | --- | --- | --- | --- | --- |
| **Layer** | **Contrast** | **Estimate** | **95% CI** | **P-value** | **Estimate** | **95% CI** | **P-value** | **Estimate** | **95% CI** | **P-value** |
| ONL | (APL-2 Monthly) - Sham pooled | 0.11 | [-0.12, 0.34] | .51 | 0.03 | [-0.15, 0.21] | .92 | 0.05 | [-0.13, 0.23] | .79 |
| ONL | (APL-2 EOM) - Sham pooled | 0.08 | [-0.15, 0.31] | .7 | 0.07 | [-0.12, 0.25] | .67 | 0 | [-0.18, 0.18] | 1 |
| ONL | (APL-2 Monthly) - (APL-2 EOM) | 0.03 | [-0.2, 0.26] | .95 | -0.04 | [-0.22, 0.15] | .89 | 0.05 | [-0.13, 0.23] | .79 |
| IS | (APL-2 Monthly) - Sham pooled | -0.13 | [-0.73, 0.47] | .86 | -0.17 | [-0.51, 0.18] | .5 | -0.03 | [-0.32, 0.26] | .96 |
| IS | (APL-2 EOM) - Sham pooled | 0.14 | [-0.47, 0.75] | .84 | 0.06 | [-0.29, 0.41] | .93 | -0.04 | [-0.34, 0.25] | .93 |
| IS | (APL-2 Monthly) - (APL-2 EOM) | -0.28 | [-0.89, 0.34] | .54 | -0.22 | [-0.57, 0.13] | .3 | 0.01 | [-0.29, 0.31] | 1 |
| OS | (APL-2 Monthly) - Sham pooled | -0.37 | [-0.81, 0.08] | .13 | -0.43 | [-0.95, 0.09] | .13 | 0.11 | [-0.2, 0.42] | .7 |
| OS | (APL-2 EOM) - Sham pooled | -0.12 | [-0.57, 0.32] | .79 | -0.46 | [-0.98, 0.07] | .1 | -0.01 | [-0.33, 0.3] | .99 |
| OS | (APL-2 Monthly) - (APL-2 EOM) | -0.24 | [-0.69, 0.21] | .42 | 0.03 | [-0.5, 0.56] | .99 | 0.12 | [-0.2, 0.44] | .64 |

** P-values were obtained using Kenward-Roger approximation to estimate the denominator degrees of freedom. P-values were adjusted within each model (i.e., the combination of layer and contour-line) using the Tukey method for comparing a family of 3 estimates.*

**Supplementary Table S5. Availability of SD-OCT data ***

| **Analysis** | **Arm** | **Overall (n)** | **Month 2 - BSL** | **Month 6 - BSL** | **Month 12 - BSL** | **Month 18 - BSL** |
| --- | --- | --- | --- | --- | --- | --- |
| Study eyes, mITT | Sham pooled | 64 | 62 | 59 | 54 | 54 |
|  | APL-2 EOM | 61 | 61 | 54 | 46 | 45 |
|  | APL-2 Monthly | 67 | 63 | 55 | 55 | 49 |
| Study eyes, PP | Sham pooled | 60 | 58 | 57 | 53 | 53 |
|  | APL-2 EOM | 57 | 57 | 52 | 44 | 43 |
|  | APL-2 Monthly | 53 | 50 | 46 | 47 | 42 |
| Study eyes, PP with the exclusion of eyes developing exudative MNV | Sham pooled | 60 | 58 | 57 | 53 | 53 |
|  | APL-2 EOM | 54 | 54 | 49 | 43 | 41 |
|  | APL-2 Monthly | 47 | 45 | 41 | 42 | 37 |
| Fellow eyes, mITT | Sham pooled | 41 | 28 | 29 | 26 | 34 |
|  | APL-2 EOM | 38 | 24 | 19 | 19 | 29 |
|  | APL-2 Monthly | 33 | 22 | 24 | 23 | 23 |

** The table shows the number (n) of available, segmented SD-OCT data used for the mixed-model analyses (after excluding scans recorded with the wrong setting or without follow-up mode). Please note that imaging data were available for some patients for baseline and later visits (e.g., baseline and month 6) but not for other visits (e.g., month 2).*

**Online-Only References**

1. Pfau M, von der Emde L, de Sisternes L, et al. Progression of Photoreceptor Degeneration in Geographic Atrophy Secondary to Age-related Macular Degeneration. *JAMA Ophthalmol*. August 2020. doi:10.1001/jamaophthalmol.2020.2914

2. Sadigh S, Cideciyan A V., Sumaroka A, et al. Abnormal thickening as well as thinning of the photoreceptor layer in intermediate age-related macular degeneration. *Investig Ophthalmol Vis Sci*. 2013;54(3):1603-1612. doi:10.1167/iovs.12-11286
